# Hinokitiol induces DNA demethylation via DNMT1 and UHRF1 inhibition in colon cancer cells

**DOI:** 10.1186/s12860-017-0130-3

**Published:** 2017-02-27

**Authors:** Jung Seon Seo, Young Ha Choi, Ji Wook Moon, Hyeon Soo Kim, Sun-Hwa Park

**Affiliations:** 1 Department of Anatomy, Institute of Human Genetics, Korea University College of Medicine, 73, Inchon-ro, Seongbuk-gu, Seoul, 02841 Republic of Korea; 2 Department of Pathology, Korea University College of Medicine, 73, Inchon-ro, Seongbuk-gu, Seoul, 02841 Republic of Korea

**Keywords:** Hinokitiol, DNA methylation, Anti-tumor activities, DNA methylation inhibitor, Colonic neoplasm

## Abstract

**Background:**

DNA hypermethylation is a key epigenetic mechanism for the silencing of many genes in cancer. Hinokitiol, a tropolone-related natural compound, is known to induce apoptosis and cell cycle arrest and has anti-inflammatory and anti-tumor activities. However, the relationship between hinokitiol and DNA methylation is not clear. The aim of our study was to explore whether hinokitiol has an inhibitory ability on the DNA methylation in colon cancer cells.

**Results:**

MTT data showed that hinokitiol had higher sensitivity in colon cancer cells, HCT-116 and SW480, than in normal colon cells, CCD18Co. Hinokitiol reduced DNA methyltransferase 1 (DNMT1) and ubiquitin-like plant homeodomain and RING finger domain 1 (UHRF1) expression in HCT-116 cells. In addition, the expression of ten-eleven translocation protein 1 (TET1), a known DNA demethylation initiator, was increased by hinokitiol treatment. ELISA and FACS data showed that hinokitiol increased the 5-hydroxymethylcytosine (5hmC) level in the both colon cancer cells, but 5-methylcytosine (5mC) level was not changed. Furthermore, hinokitiol significantly restored mRNA expression of O^6^-methylguanine DNA methyltransferase (*MGMT*), carbohydrate sulfotransferase 10 (*CHST10*), and B-cell translocation gene 4 (*BTG4*) concomitant with reduction of methylation status in HCT-116 cells.

**Conclusions:**

These results indicate that hinokitiol may exert DNA demethylation by inhibiting the expression of DNMT1 and UHRF1 in colon cancer cells.

**Electronic supplementary material:**

The online version of this article (doi:10.1186/s12860-017-0130-3) contains supplementary material, which is available to authorized users.

## Background

Epigenetic modifications are responsible for the initiation and maintenance of gene silencing. Hypermethylation of a CpG island in a promoter region is the most well-established epigenetic alteration. Aberrant promoter methylation causes transcriptional inactivation of many genes involved in tumor suppression, cell cycle regulation, apoptosis, and DNA repair. Modified DNA methylation is found in various types of cancer during carcinogenesis [1, 2]. DNA methylation is mediated by DNA methyltransferases (DNMTs), which catalyzes the transfer of methyl groups to *C* ^*5*^ of cytosine from *S*-adenosyl methionine [3]. DNMT1 maintains DNA methylation and possesses *de novo* methyltransferase activity during DNA replication, and DNMT3A and DNMT3B play an important role as *de novo* methyltransferases. DNMTs interact with transcriptional repression factors and histone deacetylases (HDACs) and thus directly causes transcription inactivation [4]. DNMT1 is recruited by replication foci via its interaction with the ubiquitin-like plant homeodomain and RING finger domain 1 (UHRF1). It was well known that UHRF1 is involved in *de novo* methylation of DNMT3A and DNMT3B and plays a pivotal role in carcinogenesis through gene silencing mechanisms and co-operating with HDAC1, which activates the DNMTs and recruited by methyl CpG binding proteins [5]. On the other hand, recent evidence demonstrates that human ten-eleven translocation (TET) enzymes have catalytic activity capable to convert 5-methylcytosine (5mC) to 5-hydroxymethylcytosine (5hmC), resulting in an initiation of DNA demethylation [6].

Currently, targeting enzymes that modify DNA methylation is considered an attractive therapeutic strategy for cancer treatment. Indeed, DNMT inhibition blocks the methylation of newly synthesized DNA strands, resulting in the reversion of the methylation status and the reactivation of silenced genes, such as tumor suppressors [7]. Several DNMT inhibitors, including 5-aza-2′-deoxycytidine (5-aza-dC), zebularine, and (−)-epigallocatechin-3-gallate (EGCG), reduce DNA methylation and re-express silenced genes. Thus, they have been suggested as potential anticancer drugs in various cancer cells *in vitro* and *in vivo*, but side effects such as DNA mutagenesis and cytotoxicity are still a cause for concern [7–9].

Hinokitiol (4-isopropyltropolone) is a component of essential oils extracted from *Chymacyparis obtusa* and has anti-infective, anti-oxidative effects, and anti-tumor activities. The anti-tumor activity of hinokitiol has been demonstrated in several types of cancer cells by inhibiting cell growth and inducing apoptosis [10–12]. However, the relevant molecular mechanisms of hinokitiol regarding anti-cancer effects are still unclear.

The goal of this study was to investigate a possible mechanism of hinokitiol on DNA methylation in human colon cancer cell lines. Our data demonstrated that hinokitiol decreased DNMT1 and UHRF1 expression and increased the level of TET1 in colon cancer cell line HCT-116. Furthermore, hinokitiol altered the methylation status of 10 hypermethylated genes in colon cancer cells and significantly reactivated the mRNA expression of O^6^-methylguanine DNA methyltransferase (*MGMT*), carbohydrate sulfotransferase 10 (*CHST10*), and B-cell translocation gene 4 (*BTG4*), which are involved in cell proliferation or biological oxidation [13–15].

## Results

### Hinokitiol inhibits colon cancer cell growth in a dose- and time-dependent manner

**Fig. 1:**
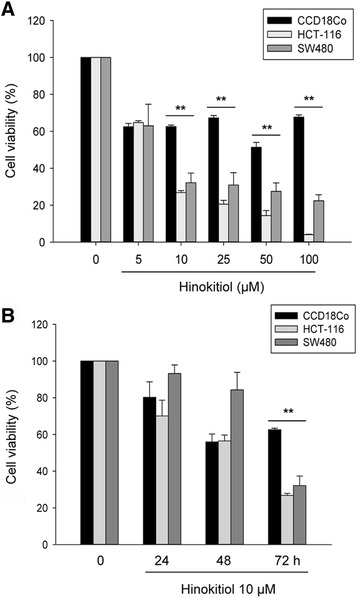
Hinokitiol inhibits the proliferation of colon cancer cells. CCD18Co, HCT-116, and SW480 cells were treated with hinokitiol at indicated concentrations for 72 h (**a**) and for periods (**b**). An equal volume of DMSO was treated as a vehicle control. Cell proliferation was measured through MTT assay. The results are representative of three different experiments and expressed as the mean ± SD and as percentage of control. ** indicates a significant difference at the level of < 0.001. Normal colon cell line: CCD18Co; colon cancer cell lines: HCT-116 and SW480

To gain insight into the anti-proliferation effects of hinokitiol in colon cancer cells, we treated HCT-116 and SW480 cells with hinokitiol of different concentrations and times. Using cell morphological observation, we found that the number of cells decreased with increasing concentrations of hinokitiol (Additional file 1A). To compare the effects of hinokitiol on the viability of colon cancer and normal colon cells, a MTT assay was performed. As shown in Fig. 1, hinokitiol affected the viability of HCT-116 and SW480 cells in dose- and time-dependent manners. In contrast, the viability of normal colon cells was maintained at concentrations over 5 μM of hinokitiol. Our data showed that hinokitiol had higher sensitivity in colon cancer cells than in normal colon cells (P < 0.001).

### Hinokitiol inhibits the expression of DNMT1 in HCT-116 cells

**Fig. 2:**
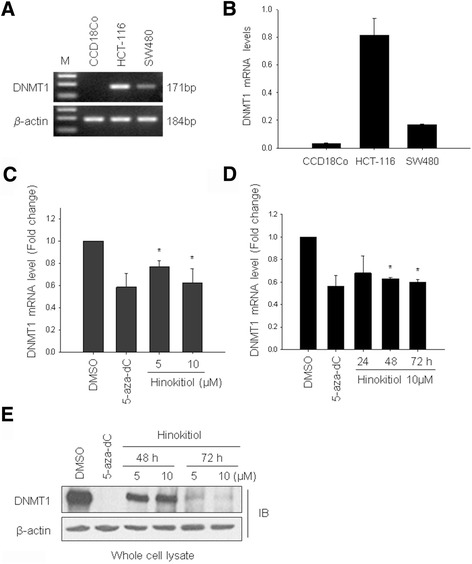
Hinokitiol decreases DNMT1 mRNA and protein expression in HCT-116 cells. The level of DNMT1 mRNA was determined in HCT-116 and SW480 cells using RT-PCR (**a**) and quantitative real-time PCR (qRT-PCR) (**b**). PCR products were gel-run in 2% agarose and visualized in ultraviolet (UV). *β*-actin was used as a quantitative control. The quantitative DNMT1 mRNA level was measured using qRT-PCR after hinokitiol treatment at indicated concentrations and times in HCT-116 cells (**c** and **d**). Total protein was isolated from cells treated with indicated concentrations of hinokitiol for 72 h and western blotting was performed to detect DNMT1 expression (**e**). The results were representative of three independent experiments. Cells treated with DMSO and 5-aza-dC were used as negative and positive controls, respectively. The *β*-actin was used as a loading control. * indicates a significant difference at the level of < 0.05. M, 100 bp DNA ladder

The expression patterns of DNMT1 in normal colon cells and colon cancer cells were analyzed by RT-PCR and qRT-PCR. In Fig. 2a and b, DNMT1 mRNA is highly expressed in colon cancer cells, especially in HCT-116 cells, while only slightly expressed in CCD18Co cells. To gain insight into the role of hinokitiol on DNA methylation, we measured DNMT1 expression after the hinokitiol treatment. HCT-116 cells were exposed to the indicated concentrations of hinokitiol for up to 72 hrs, and their DNMT1 expression was analyzed every 24 h using qRT-PCR. We found that hinokitiol reduced DNMT1 mRNA expression in time- and dose-dependent manners (P < 0.05) (Fig. 2c and d). In addition, the effect of hinokitiol on the expression of DNMT1 protein was confirmed using Western blotting. As shown in Fig. 2e, hinokitiol (5 and 10 μM) treatment decreased the protein level of DNMT1 in a time-dependent manner. Furthermore, the expression levels of DNMT1 mRNA and protein with 10 μM of hinokitiol for 72 h were similar to that of the 5-aza-dC treatment. We also showed the decreased expression of DNMT1 protein in SW480 cells treated with hinokitiol for 72 h (Additional file 1B). These results suggest that hinokitiol may be a promising agent for DNMT1 inhibition in colon cancer cells.

### Hinokitiol inhibits the expression of UHRF1 in HCT-116 cells

**Fig. 3:**
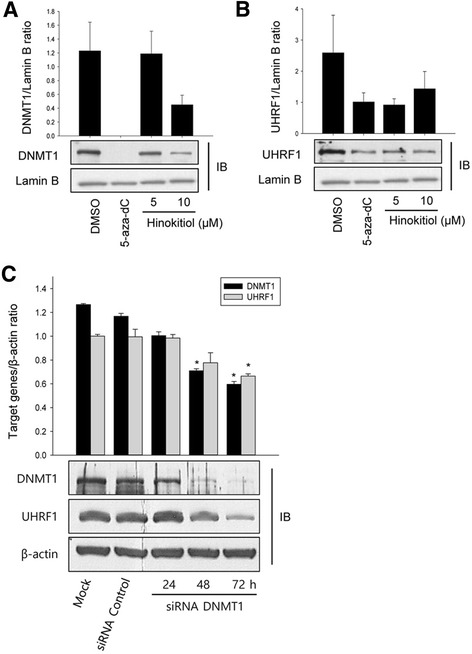
Hinokitiol decreases UHRF1 protein expression in HCT-116 cells. HCT-116 cells were treated with indicated concentrations of hinokitiol for 72 h. Nuclear protein was extracted from the cells for western blot analysis with indicated antibodies (**a**-**b**). Lamin B was used for loading control. HCT-116 cells were seeded into culture plates. After 24 h cultivation, cells were transiently transfected with siRNA DNMT1 and siRNA Control for 24, 48, 72 h, collected, and total protein was extracted for western blot analysis using indicated antibodies (**c**). Experiments were done twice and representative blots were shown. The expression level of each protein was quantified with the Image Studio Lite program, using Lamin B as a loading control. The histogram shows the quantification expressed as ratio of the intensity of target gene/Lamin B. * indicates a significant difference at the level of < 0.05

UHRF1 forms a complex with DNMT1 to maintain DNA methylation. In order to investigate the involvement of hinokitiol on UHRF1, the level of UHRF1 protein was measured in nuclear extracts of HCT-116 cells. Like in Fig. 2e, hinokitiol decreased level of DNMT1 protein (Fig. 3a). In addition, the level of UHRF1 protein was reduced in HCT-116 cells exposed to 5 and 10 μM of hinokitiol, whereas the control cells were not (Fig. 3b). Furthermore, knock-down DNMT1 expression by siRNA reduced UHRF1 protein level (Fig. 3c). These results suggest that hinokitiol may be associated with DNA demethylation pathways in HCT-116 cells.

### Hinokitiol enhances TET1 activity in HCT-116 cells

**Fig. 4:**
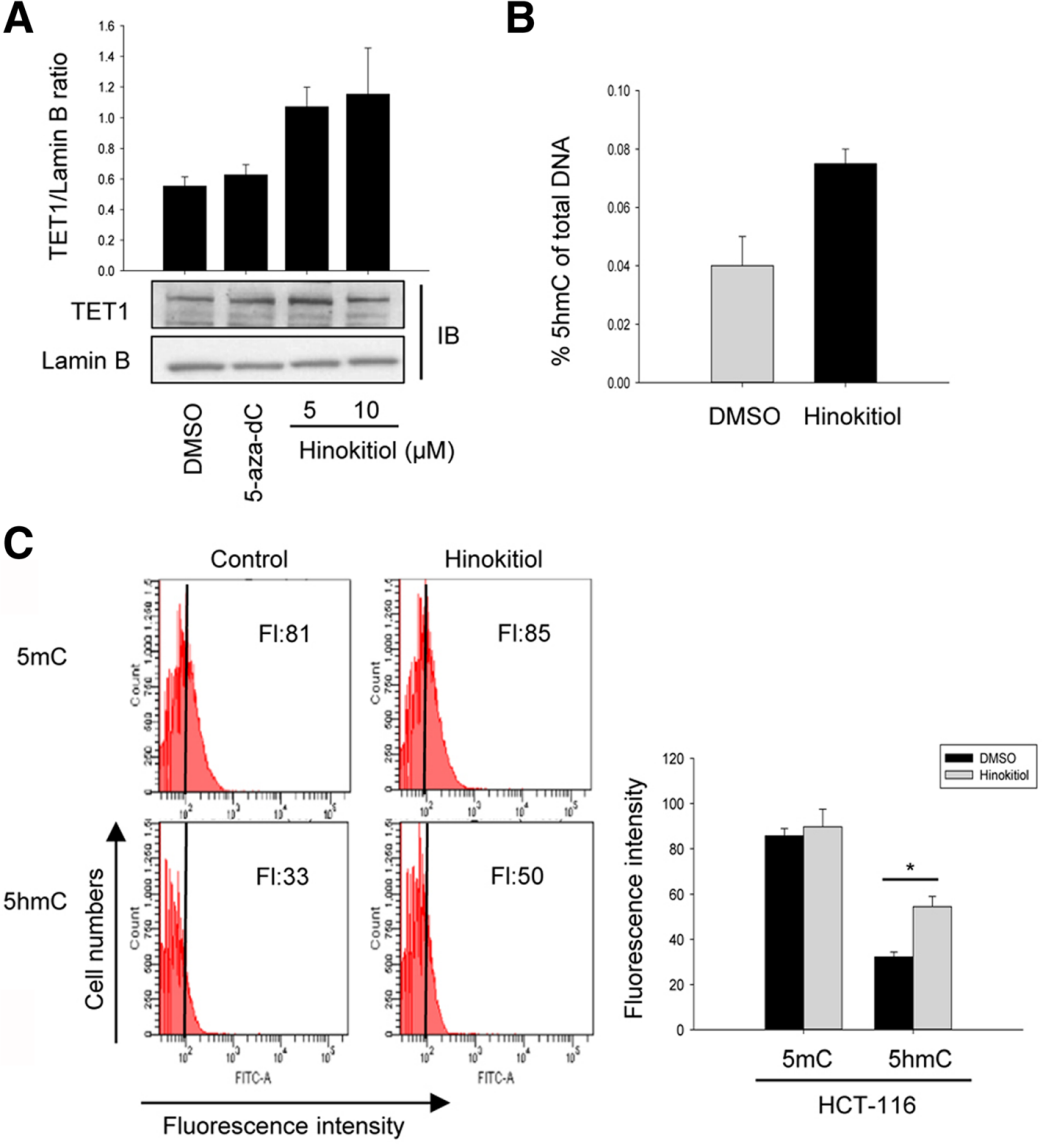
Hinokitiol increases TET1 expression via enhancement of 5hmC level in HCT-116 cells. HCT-116 cells were treated with 5 and 10 μM of hinokitiol for 72 h and nuclear protein isolated from the cells was used to detect TET1 expression using western blot analysis (**a**). The expression level of each protein was quantified with the Image Studio Lite program, using Lamin B as a loading control. The histogram shows the quantification expressed as ratio of the intensity of target gene/Lamin B. Experiments were done twice and representative blots were shown. Contents of 5hmC measured from cells treated with 10 μM of hinokitiol for 72 h using ELISA-based methylflash hydroxymethylated DNA quantification kit (**b**). The levels of 5mC and 5hmC were confirmed using flow cytometry analysis (**c**). All data are representative of three independent experiments performed in duplicate. The results were representative of three independent experiments. Data are the means ± SE of results from at least three independent experiments. * indicates a significant difference at the level of < 0.05

To confirm the effect of hinoikitiol on DNA demethylation, TET1 protein expression in nuclear fraction of cells was evaluated by Western blotting. Hinokitiol increased TET1 protein expression in a dose-dependent manner and its level was higher than that of 5-aza-dC treatment (Fig. 4a). In addition, the alteration of 5hmC level by hinokitiol treatment was assessed by using ELISA in HCT-116 cells. Pretreatment with 10 μM hinokitiol increased the 5hmC level of total DNA about 1.87-fold (from 0.040 to 0.075) compared to the control (Fig. 4b). TET1 activity was also analyzed by measuring the levels of 5mC and 5hmC using FACS analysis. Hinokitiol treatment induced a significant enhancement of 5hmC level (fluorescence intensity, FI:50) (P < 0.05). The 5mC level was not affected in either treatment (Fig. 4c). These results indicate that hinokitiol may cause DNA demethylation through the downregulation of DNMT1 as well as the upregulation of TET1 without reducing the level of 5mC in colon cancer cells.

### Hinokitiol restores the mRNA expression of *MGMT*, *BTG4*, and *CHST10* via demethylation

**Fig. 5:**
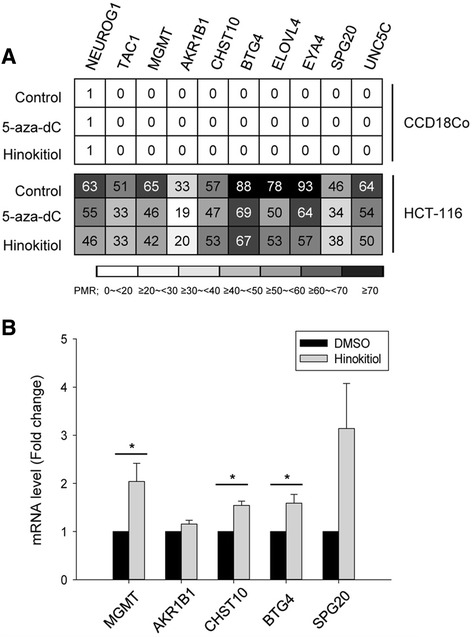
Hinokitiol reduces methylation status and restores mRNA expression of *MGMT*, *CHST10*, and *BTG4* genes. The effects of hinokitiol on the methylation status of hypermethylated genes in HCT-116 cells compared with CCD18Co, were assessed using QMSP. The ratio of methylation intensity was determined by the percentage of methylated reference (PMR). Cells treated with DMSO or 5-aza-dC at the same condition were used as negative and positive controls, respectively (**a**) The mRNA expressions of *MGMT*, *AKR1B1*, *CHST10*, *BTG4*, and *SPG20* genes were measured using qRT-PCR (**b**). Genomic DNA and total RNA were extracted from cells treated with 10 μM of hinokitiol for 72 h. Data are the means ± SE of results from at least three independent experiments. * indicates a significant difference at the level of < 0.05

To verify the effect of demethylation and restoration of hinokitiol on silenced genes resulting from DNA methylation, the levels of methylation and mRNA of three CIMP markers and seven candidate genes in colon cancer cells were analyzed by using QMSP and qRT-PCR, respectively. In our previous study, we observed that three CIMP markers (*NEUROG1, TAC1,* and *MGMT*) and seven novel methylation candidate genes (*AKR1B1, CHST10, BTG4, ELOVL4, EYA4, SPG20*, and *UNC5C*) were hypermethylated with the loss of the respective mRNA expression in HCT-116 cells but not in CCD18Co cells [16]. Here, the methylation status of these genes was reduced after treating the cells with 10 μM of hinokitiol for 72 h, though not completely reduced, and hinokitiol-reversed methylation levels of those genes were similar to that produced by a 5-aza-dC (Fig. 5a). Moreover, hinokitiol significantly reactivated the mRNA expression of *MGMT, CHST10,* and *BTG4* (P < 0.05) (Fig. 5b).

## Discussion

The key findings of this study are that there were significant differences in sensitivity to hinokitiol between both colon cancer cells and normal colon cells. This result was similar to those of other reports showing that hinokitiol inhibits cell viability in colon cancer cells (HCT116 and SW620) but not in normal colon cells (CCD112CoN) [12]. Importantly, we demonstrated that hinokitiol decreased the protein expression of DNMT1 as well as UHRF1 in HCT-116 cells, and its effect was similar with that of 5-aza-dC. Hinokitiol is a natural tropolone-based monoterpenoid which is found in *cupressacceous* plants. It has been shown that hinokitiol possesses potent anti-tumor effects in various cancer cell lines, including colon cancer, lung adenocarcinoma, breast cancer, and melanoma cells, by inducing cell cycle arrest or apoptosis [12, 17, 18]. However, previous studies have focused on elucidating the molecular mechanism of hinokitiol-induced anticancer effects through apoptosis pathways, and the mechanisms underlying its effects are not yet fully understood. In the present study, our results demonstrated that hinokitiol has potential as a novel DNMT inhibitor and could be associated with DNA methylation and thus provide hinokitiol as new therapeutic candidate of colon cancer.

Epigenetic alterations, including DNA methylation and histone modifications, play crucial roles in carcinogenesis and show possible targets for cancer treatment and prevention. DNMT1 is most abundantly found in mammalian cells. Increased DNMT expression has been proposed as a mechanism for the increased methylation that occurs in the promoter region of tumors. In DNA methylation, the role of DNMTs is more complex and may involve changes in the expression of mRNA or protein. Previous studies have reported that DNMT1 inhibition correlates with reduction in tumorigenicity and increased expression of tumor suppressor genes, such as *p16* ^*INK4a*^ or *p14* ^*ARF*^ [19, 20]. Thus, DNMT1 represents one of the most attractive targets for development of anticancer drugs. For example, azacytidine and decitabine are well known as DNMT inhibitors and the most successful epigenetic modulators. Zebularine is another DNMT inhibitor with concomitant inhibitory activity towards cytidine deaminase. EGCG has dual actions involving both DNA demethylation and posttranslational histone modifications in cancer cells [7, 9, 21, 22]. However, there are still restrictions because of their toxicity and poor stability or lack of information linked to the possible clinical applications.

In the present study, we determined that hinokitiol decreased UHRF1 proteins, which was similar or lower with those of 5-aza-dC in HCT-116 cells and demonstrated that such UHRF1 inhibition was caused by DNMT1 reduced via transient transfection of siRNA DNMT1. It has been reported that increased UHRF1 is associated with cellular proliferation and has observed in various types of cancers such as colorectal cancer. In addition, UHRF1 binds to methylated promoters of many tumor suppressor genes by forming complexes with DNMTs and HDAC1, resulting ultimately in the formation of cancer [23, 24]. Therefore, our results indicate that hinokitiol may directly regulate the expression of DNA methylation-related genes and may inhibit cellular proliferation in colon cancer. We also measured the expression of HDAC1 protein to investigate whether hinokitiol could affect histone deacetylation. Incubation of cells to hinokitiol for 72 h resulted in a dose-dependent decrease of HDAC1 protein levels in the same cells, and its expression was lower than that of 5-aza-dC (Additional file 2).

Ten-eleven translocation (TET) enzymes are have been identified as key players in demethylation of cytosine. TET family genes are frequently observed in human cancers [25]. Our data demonstrated that hinokitiol increased the expression of TET1 protein well known as DNA demethylation initiator in HCT-116 and SW480 cells (Additional file 3) but did not affect to the 5mC level. TET1 is an iron-dependent α-ketoglutarate dioxygenase enzyme, and both fully methylated and hemimethylated DNA in a CG or non-CG context can serve as substrates for it. TET1 is responsible for converting 5mC to 5hmC methylcytosine because of causing demethylation. Inactivation of TET1 is associated with aberrant DNA methylation in cancers. TET1 methylation is connected with CpG island methylator phenotype (CIMP) in colorectal cancer [26, 27]. On the other hand, several studies reported that various chemical agents such as *GSH*, *vitamin B1*, and *vitamin E*, appeared not to affect 5mC oxidation patterns despite DNA demethylation effects via an increase of 5hmC level in cultured cells [28, 29]. We also observed whether hinokitiol affects both TET2 and TET3 protein levels. Our data showed that hinokitiol had little effect on the expression of TET3 protein in HCT-116 and SW480 cells (Additional file 4). On the other hand, TET2 protein expression did not detected in both cells (data not shown). It might be because TET2 has a higher expression level in hematological cells than TET1 or TET3 and is recruited to genomic DNA by distinct CXXC domain-independent mechanism unlike TET1 and TET3 [30]. Therefore, our results suggest that hinokitiol may cause TET1-mediated DNA modifications in colon cancer cells without 5mC reduction.

Furthermore, our data demonstrates that hinokitiol alters the methylation status of hypermethylated genes in HCT-116 cells. Three CIMP markers and seven new methylation candidates, which were reported in our previous study [16], were selected randomly and assessed by using QMSP for this study. In this experiment, hinokitiol reduced methylation status of ten genes and induced the significant restoration of *MGMT*, *CHST10*, and *BTG4* mRNA expression. *MGMT* is a DNA repair enzyme involved in direct repair of alkylation damage products. *CHST10* is also known to inhibit the invasiveness of melanoma cells, and *BTG4* has anti-proliferative properties [13, 15, 31]. The evidence for promoter methylation patterns of those genes has been shown in various cancers, including colon cancer [31–33]. Further studies are required to clarify these relationships, particularly that hinokitiol induces effectively the reactivation of epigenetic-silencing genes in colon cancer cells through combination treatment with HDAC inhibitors or chemotherapeutic drugs, including 5-FU and oxaliplatin.

## Conclusions

This study has demonstrated the relationship between hinokitiol and DNA methylation in colon cancer cells and provided evidence for the potential of hinokitiol as a novel DNMT1 inhibitor for colon cancer treatment.

## Methods

### Cell culture and chemical treatment

The human colon cancer cell lines (HCT-116 and SW480) and a normal colon cell line (CCD18Co) were purchased from Korean Cell Line Bank (KCLB, Seoul, Korea) and the American Type Culture Collection (ATCC, Manassas, VA, USA). CRC cell lines were maintained in RPMI-1640 medium (WELGENE, Daejeon, Korea), and CCD18Co cell line was maintained in Eagle’s minimum essential medium (MEM, WELGENE) containing 10% FBS and 1% penicillin-streptomycin in a 5% CO2 atmosphere at 37 °C. Hinokitiol (*β*-thujaplicin) and 5-aza-dC were dissolved in dimethyl sulfoxide (DMSO). All chemicals were purchased from Sigma-Aldrich (St. Louis, MO, USA). As a control, 10 μM of 5-aza-dC was added freshly every 24 h for 3 days.

### Cell viability assay and morphologic

Cell viability was evaluated using MTT (3-(4, 5-Dimethylthiazol-2-yl)-2, 5-diphenyltetrazolium bromide) assay. Briefly, cells were cultured in 96-well plates for 24 h and then incubated with different concentrations of hinokitiol (5–100 μM) for 72 h in a 5% CO_2_ incubator at 37 °C. At the indicated times, 0.5 mg/ml MTT solution was added to the wells. After a further 3 h of incubation, the supernatant was discarded, and DMSO was added to the plates. The color intensity was measured at 570 nm using microplate reader (SpectraMax Puls 384, Molecular Devices, USA). The cell viability of each sample was presented as a percentage of the viability of culture treated with control. Cells treated with DMSO were used as a control, which was considered to be 100% viable. These tests were performed on all samples at least three times in the same assay. Changes in the morphology of cells cultured with hinokitiol were observed by using the EVOS® Image Systems (Thermo Fisher Scientific, Hudson, NH, USA).

### RNA extraction and reverse transcription (RT)-PCR

RNA was isolated from cells using the QIAzol lysis reagent (Qiagen, Hilden, Germany), according to the manufacturer’s instructions. cDNA synthesis was performed in mixtures containing 2 μg of RNA, oligo-dT, and AMV-reverse transcriptase (Promega, Madison, WI), following the manufacturer’s instructions. PCR was performed using a SureCycler 8800 Thermal Cycler (Agilent Technologies, Santa Clara, CA, USA). The primers used in the PCR reactions are summarized in Table 1. The PCR program was initiated at 95 °C for 10 min, followed by 35 cycles of 95 °C for 15 s, 60 °C for 20 s, and 72 °C for 45 s. The amplified products were separated on a 2% agarose gel, stained with Safe Shine Green (Biosesang, Korea), and visualized using BioDoc-It Imaging Systems (An Analytik Jena Company, CA, USA). *β*-Actin was used to normalize the amount of cDNA.

**Table 1:** Primers for mRNA expression

Genes	Primer sequences (5′ → 3′)	Annealing temp. (°C)	Product size (bp)
DNMT1	F:AGACTACGCGAGATTCGAGTC	60	171
	R:TTGGTGGCTGAGTAGTAGAGG		
MGMT	F:ACCGTTTGCGACTTGGTACT’	54	268
	R:CGGGGAACTCTTCGATAGCC		
AKR1B1	F:CCCATGTGTACCAGAATGAGAA’	58	362
	R:CTGGAGATGGTTGAAGTTGGAG’		
CHST10	F:GTGTGATTGGACACCACGAG’	58	176
	R:ATACAGGCGTCGGATGTCTC’		
BTG4	F:GCAAGGAACCTCGTGTCATT	58	159
	R:GCGAGCCATGGTAAGTGTTT’		
SPG20	F:CCAACTGGAACAGAGCAGAAG’	58	160
	R:TTAGCCCTTTTTCCACGTTTT’		
*β*-actin	F:AGAGCTACGAGCTGCCTGAC	60	184
	R:AGCACTGTGTTGGCGTACAG		

### Quantitative real-time PCR (qRT-PCR)

To detect the mRNA level of DNMT1 and 10 hypermethylated genes in HCT-116 cells, qRT-PCR was performed using ABI 7500 Real-Time PCR system (Applied Biosystems, Foster City, CA, USA). The sets of primers indicated (Table 1) and primer sequences were designed using Primer3 ver. 0.4.0 (http://primer3.ut.ee/). The experiments were repeated at least three times, and each sample was analyzed in duplicate. The relative amount of target genes was normalized by the amount of *β*-actin used as the internal control in the same sample and described as the ratio of each target gene/*β*-actin. The cDNA of a known concentration was prepared by serial dilutions, which were used as the standard curve for quantification.

### Genomic DNA extraction

Genomic DNA was extracted using the Wizard genomic DNA purification kit (Promega), according to the manufacturer’s recommendations. The cells were lysed in 600 μl of nuclei lysis solution, RNase solution was added, and the mixture was incubated at 37 °C for 30 min. The samples were mixed with 200 μl of protein precipitation solution and centrifuged at 13,200 g for 5 min at room temperature. The supernatants were transferred to fresh tubes, and the genomic DNA was precipitated with isopropanol and washed with 70% ethanol. The DNA eluted in 800 μl of DNA rehydration solution was quantified using a NanoDrop ND-1000 spectrophotometer (Thermo Fisher Scientific).

### Western blot analysis

To extract total protein, cells were lysed in RIPA buffer containing protease inhibitors (15 mM PMSF, 1 mM NaF, and 1 mM Na3VO4) and stored at −80 °C until use. In addition, a nuclear protein was isolated from cells using the EpiQuik nuclear extraction kit (Epigentek Group Inc), according to the manufacturer’s instructions. The supernatants were collected as total protein and nuclear protein extracts, respectively. Aliquots of the supernatants (50 μg of total protein or nuclear extracts) were subjected to sodium dodecyl sulfate polyacrylamide gel electrophoresis (SDS-PAGE). The proteins were separated and then transferred onto a nitrocellulose membrane (GE Healthcare Life Science, Pittsburgh, PA, USA). The membranes were incubated with anti-DNMT1 (sc-20701), UHRF1 (Abcam, Cambridge, MA, USA), TET1, TET2, TET3, Lamin B (Santa Cruz Biotechnology, CA, USA), and *β*-actin (Sigma-Aldrich) antibodies. After washing, the membranes were incubated with horseradish peroxidase-conjugated secondary antibodies (Santa Cruz Biotechnology). The bound antibodies were visualized using the SuperSignal West Dura Extended Duration Substrate (Thermo-Fisher Scientific). *β*-actin for total protein and Lamin B for a nuclear protein were used to confirm comparable loading. The expression level of each protein was quantified with the Image J program using Lamin B or *β*-actin as a loading control.

### Transient transfection of DNMT1 small RNA interference (siRNA)

Cells were seeded at 0.3×10^5^ cells/well in 6-well plates and allowed to reach approximately 60% confluence on the day of transfection. The siRNA constructs synthesized were: a mismatched siRNA control and an siRNA against DNMT1 (Cosmogenetech, Seoul, Korea). Cells were transfected with 50–100 nM siRNA using lipofectamine 3000 based on the manufacturer’s protocol. Cells were harvested and examined by qRT-PCR and western blot analysis at 24, 48, and 72 h after transfection.

### Quantification of 5mC and 5hmC

For quantification of 5hmC, the ELISA-based methylflash hydroxymethylated DNA quantification kit (Epigentek Group Inc., NY, USA) was used. The initial incubation time was 90 min, and the final developing time was 10 min. The absolute quantification of standard curves was generated by plotting the concentration of the positive control supplied with the assay against the optical density at 450 nm after performing the assay. The amount of 5hmC was assessed by absolute quantification. For detection of the levels of 5mC and 5hmC by flow cytometry, cells were washed with PBS supplemented with 0.1% Tween-20 and 1% bovine serum albumin (PBST-BSA) and fixed with 0.25% paraformaldehyde at 37 °C for 10 min and 88% methanol at −20 °C for 1 h. Cells were permeated with 10% Triton-X 100 at room temperature for 10 min and then treated with 2 N HCL at 37 °C for 30 min and neutralized with 0.1 M sodium borate (pH 8.5). The cells were blocked with 10% FBS in PBST-BSA at 37 °C for 20 min, incubated with anti-5mC and anti-5hmC (Active Motif, Carlsbad, CA, USA) antibodies at 37 °C for 50 min, followed by staining with Cy3- or Alexa 488-conjugated secondary antibodies (Jackson ImmunoResearch Laboratories, PA, USA). The cells were washed with PBS twice and analyzed by using a flow cytometer (FACS Canto II, BD-Science, San Jose, CA, USA).

### Bisulfite treatment and quantitative real-time methylation-specific PCR (QMSP)

Bisulfite modification of DNA was performed using the EpiTect Fast Bisulfite Conversion kits (Qiagen), following the manufacturer’s directions. The reaction was prepared by mixing 85 μl of bisulfite solution and 35 μl of DNA protect buffer in PCR tubes at room temperature. The modified DNA was purified using Qiaquick gel extraction kit (Qiagen) and eluted with water. The methylation status of *NEUROG1*, *TAC1*, *MGMT*, *AKR1B1*, *CHST10*, *BTG4*, *ELOVL4*, *EYA4*, *SPG20*, and *UNC5C* genes was determined by QMSP using ABI 7500 Real-time PCR Systems (Applied Biosystems). Primers for QMSP were designed for potential CpG islands near the translation start site using the NCBI database and summarized in Table 2. Methylation primers were designed using MethPrimer software (http://www.urogene.org/methprimer/). The PCR program was initiated at 95 °C for 5 min, followed by 40 cycles of 95 °C for 15 s and 54–60 °C for 1 min. The experiments were repeated at least three times, and each sample was analyzed in duplicates. Relative quantification of the amplified gene levels in bisulfite-converted genomic DNA sample was performed by measuring the threshold cycle (CT) values of target genes and *β*-actin. The relative amount of target genes was normalized by the amount of *β*-actin used as the internal control in the same sample and described as the ratio of each target gene/*β*-actin. The bisulfite-converted genomic DNA of a known concentration was prepared by serial dilutions, which were used as the standard curve for quantification. The modified genomic DNA by CpG methyltransferase M. *Sss*I (NEB, Ipswich, MA, USA) was used as a positive control. DNA methylation according to M.*Sss*I was verified using the restriction enzyme B*stU*I (NEB).

**Table 2:** Primers for quantitative methylation-specific PCR (QMSP)

Gene	Primer sequences (5′ → 3′)	Annealing temp. (°C)	Product size (bp)	Location	Gene bank
NEUROG1	F:TATGTAAATATTCGGGCGTTGTAC	58	199	(−155 ~ +43)	NC_000005.9
	R:GATCTCCTAAATAATATCGCCGAC				
TAC1	F:TTAGATTTGTAGACGGAAGTAGGTC	58	139	(−135 ~ +3)	NC_000007.13
	R:GTAATTAAAAATTTCCGAAACGAT				
MGMT	F:GTTTGTATTGGTTGAAGGGTTATTT	58	109	(−292 ~ −184)	NC_000010.10
	R:CTAAAACAATCTACACATCCTCACT				
AKR1B1	F:CGGAAGAAGTATTTTCGTCGA	58	166	(−120 ~ +45)	NC_000007.13
	R:CAATACGATACGACCTTAACCG				
CHST10	F:TTTTGTAGCGGTAGAAAGGGAGATTCG	58	198	(−125 ~ +72)	NC_000002.11
	R:GACTTTAAAAACCAAAACGCCGAC				
BTG4	F:GTATAATACGCGTAGTTGGGTTAGC	58	171	(−422 ~ −252)	NC_0000011.9
	R:AAAAAAACGAAAAAAACCTAAACG				
ELOVL4	F:GAGTTTAGGTGTTTCGTTTTCGTTC	58	114	(−158 ~ −42)	NC_000006.11
	R:CCTCCCTCCCTAATATTAAAACTCG				
EYA4	F:GTTATTCGAGGTTAAATAAAAACGG	58	149	(−198 ~ −50)	NC_000006.11
	R: ACTTACGCAAAAAAATAAAACGAA				
SPG20	F:GTCGAGTAGTCGACGTGGTC	58	168	(−246 ~ −78)	NC_000013.10
	R:AATAATACGTAAAAAAACGTCCGTC				
UNC5C	F:GTTTAGGTTTGGCGTATCGC	58	219	(−463 ~ −245)	NC_000004.11
	R:GCCAAAAAAACGTAAAAAACG				
*β*-actin	F:TGGTGATGGAGGAGGTTTAGTAAGT	58	132	(−1645 ~ −1513)	NC_000007.13
	R: AACCAATAAAACCTACTCCTCCCTTAA				

### Statistical analysis

Results are presented as means ± SE. For statistical analyses, Student’s t-test for independent samples was used. A value of P < 0.01 or < 0.05 was considered to indicate statistical significance. All analyses were performed using SigmaPlot12.0.

## Additional files

Additional file 1: Hinokitiol affects cell morphology and DNMT1 protein expression in colon cancer cells. HCT-116 cells were treated with 10 μM of hinokitiol and cultured for 72 h. Changes in cell morphology was observed under the phase contrast microscope by using EVOS Image System (a). SW480 cells were treated with 10 μM of hinokitiol for 72 h. Nuclear protein was isolated from the cells and 50 μg of protein was separated on SDS-PAGE. After then, western blot analysis was performed with anti-DNMT1 and anti-Lamin B antibodies. Lamin B was used for loading control (b). Cells treated with DMSO or 5-aza-dC were used as negative and positive controls, respectively. (JPG 289 kb)
Additional file 2: Hinokitiol decreases HDAC1 protein expression in HCT-116 cells. HCT-116 cells were treated with indicated concentrations of hinokitiol for 72 h. Nuclear protein was extracted from the cells for western blot analysis with anti-HDAC1 and anti-Lamin B antibodies (Upper panel). Lamin B was used for loading control. Experiments were done twice and representative blots were shown. The expression level of each protein was quantified with the Image Studio Lite program, using Lamin B as a loading control. The histogram shows the quantification expressed as ratio of the intensity of target gene/Lamin B (lower panel). (JPG 170 kb)
Additional file 3: Hinokitiol enhances of 5hmC level in SW480 cells. SW480 cells were treated with 5 and 10 μM of hinokitiol for 72 h and nuclear protein was isolated. Contents of 5hmC was measured using ELISA-based methylflash hydroxymethylated DNA quantification kit (a). The levels of 5mC and 5hmC were confirmed using flow cytometry analysis (b). All data are representative of three independent experiments performed in duplicate. The results were representative of three independent experiments. Data are the means ± SE of results from at least three independent experiments. (JPG 246 kb)
Additional file 4: Hinokitiol has no affect TET3 protein expression in colon cancer cells. Colon cancer cells (HCT-116 or SW480) were treated with 10 μM of hinokitiol for 72 h. Nuclear protein was extracted from the cells and then western blot analysis was performed with anti-TET3 and anti-Lamin B antibodies. Lamin B was used for loading control. Experiments were done twice and representative blots were shown. Cells treated with DMSO or 5-aza-dC were used as negative and positive controls, respectively. (JPG 102 kb)

## Abbreviations

5-aza-dC: 5-aza-2′-deoxycytidine
5-hmC: 5-hydroxymethylcytosine
5-mC: 5-methylacytosine
AKR1B1: Aldo-keto reductase 1
BTG4: B-cell translocation gene 4
CHST10: carbohydrate sulfotransferase 10
CpG: Cytosine-phosphate-guanine
DMSO: Dimethyl sulfoxide
DNMT: DNA methyltransferease
ELOVL4: Elongation of very long chain fatty acids protein 4
EYA4: Eyes absent homolog 4
GSH: Glutathione
HDAC: Histone deacethylase
MGMT: O^6^-methylguanine DNA methyltransferase
NEUROG1: Neurogenin-1
SPG20: Spastic paraplegia 20
TAC1: Protachykinin-1
TETs: Ten-eleven translocation proteins
UHRF1: Ubiquitin-like plant homeodomain and RING finger domain 1
UNC5C: Unc-5 homolog C
