# Supplementary figures and images for: Hinokitiol induces DNA demethylation via DNMT1 and UHRF1 inhibition in colon cancer cells
Source: BMC Cell Biol. 2017 Feb 27;18:14. doi: 10.1186/s12860-017-0130-3 (PMC5327573; doi:10.1186/s12860-017-0130-3)

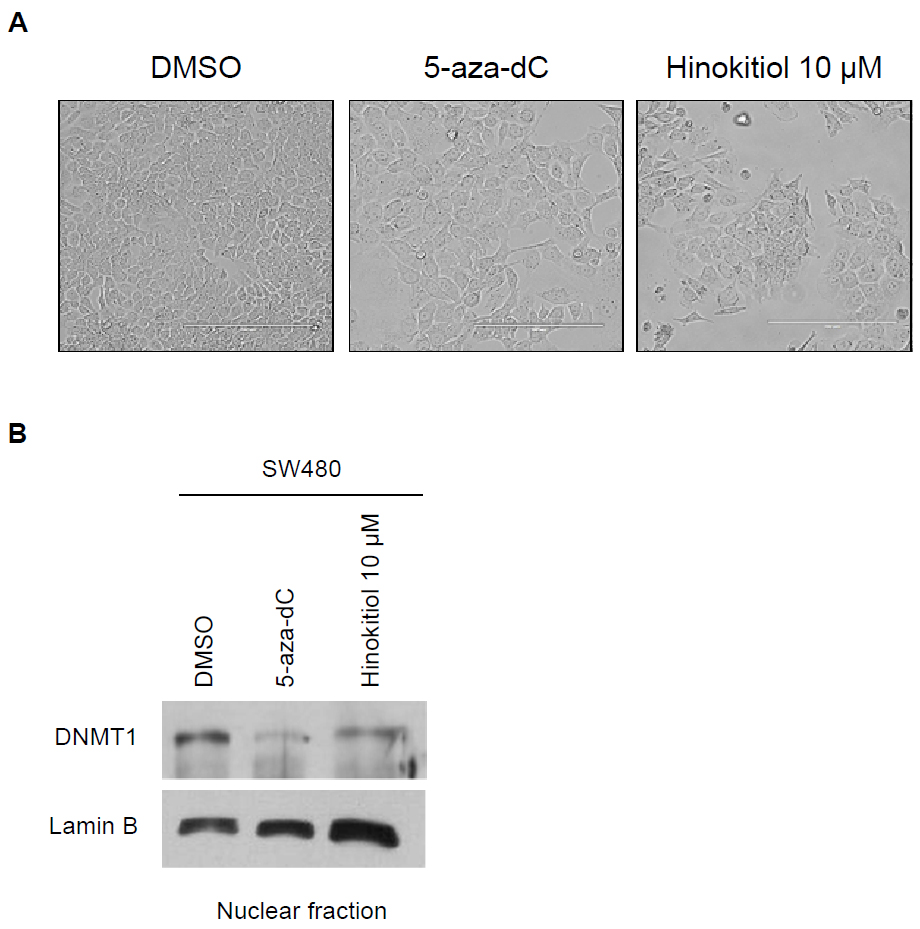

Supplement: Additional file 1: — Hinokitiol affects cell morphology and DNMT1 protein expression in colon cancer cells. HCT-116 cells were treated with 10 μM of hinokitiol and cultured for 72 h. Changes in cell morphology was observed under the phase contrast microscope by using EVOS Image System (a). SW480 cells were treated with 10 μM of hinokitiol for 72 h. Nuclear protein was isolated from the cells and 50 μg of protein was separated on SDS-PAGE. After then, western blot analysis was performed with anti-DNMT1 and anti-Lamin B antibodies. Lamin B was used for loading control (b). Cells treated with DMSO or 5-aza-dC were used as negative and positive controls, respectively. (JPG 289 kb) [file 12860_2017_130_MOESM1_ESM.jpg]

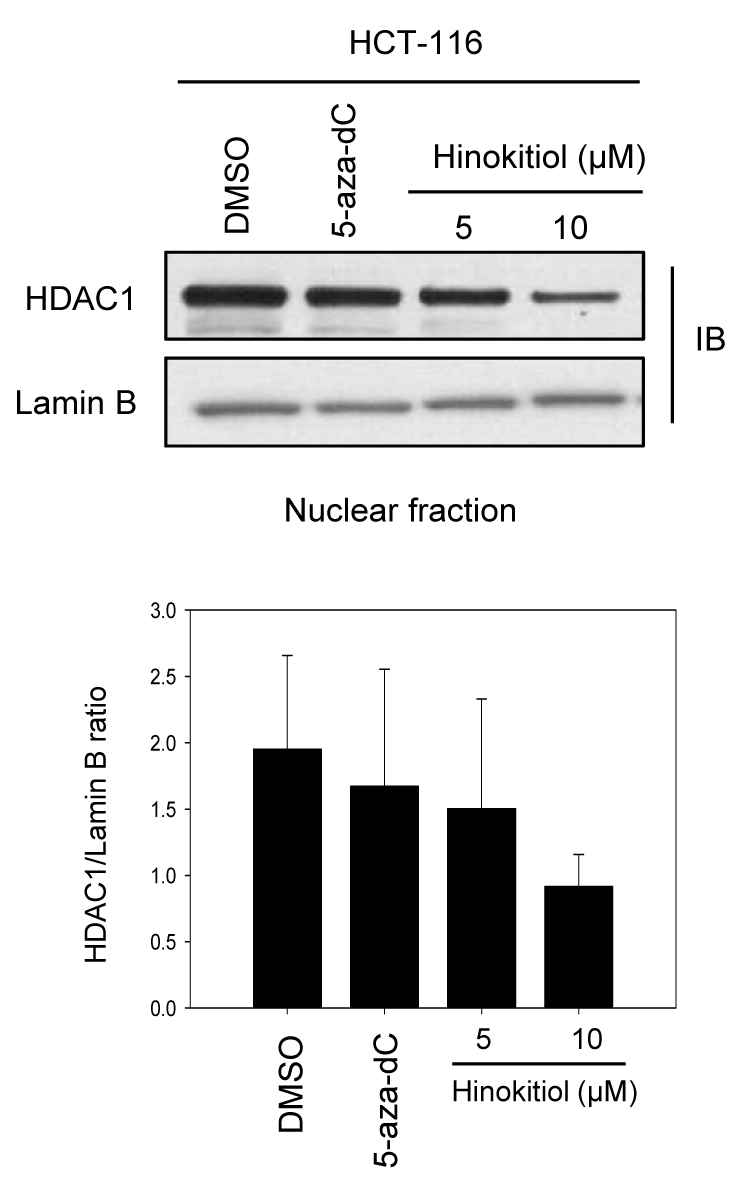

Supplement: Additional file 2: — Hinokitiol decreases HDAC1 protein expression in HCT-116 cells. HCT-116 cells were treated with indicated concentrations of hinokitiol for 72 h. Nuclear protein was extracted from the cells for western blot analysis with anti-HDAC1 and anti-Lamin B antibodies (Upper panel). Lamin B was used for loading control. Experiments were done twice and representative blots were shown. The expression level of each protein was quantified with the Image Studio Lite program, using Lamin B as a loading control. The histogram shows the quantification expressed as ratio of the intensity of target gene/Lamin B (lower panel). (JPG 170 kb) [file 12860_2017_130_MOESM2_ESM.jpg]

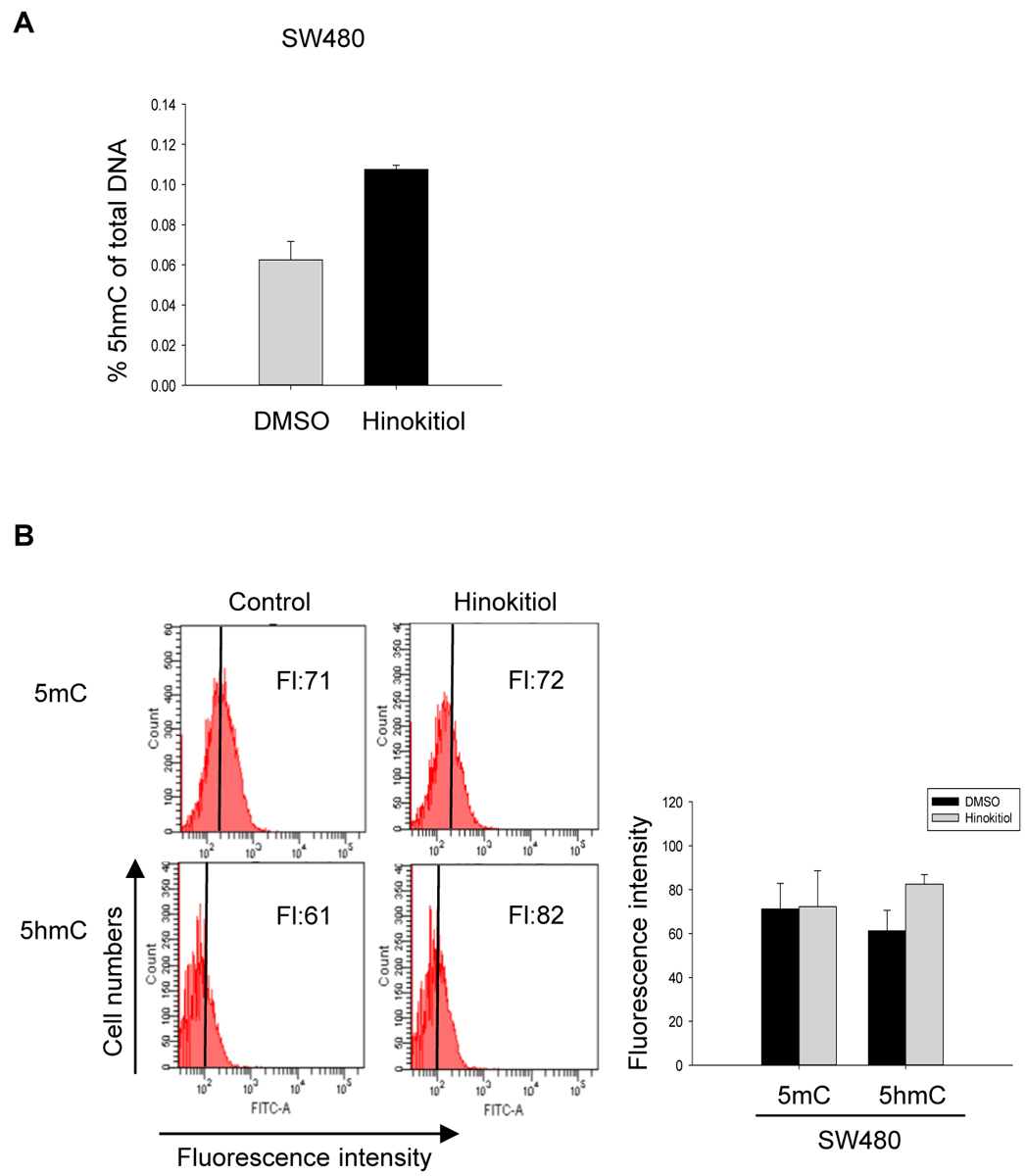

Supplement: Additional file 3: — Hinokitiol enhances of 5hmC level in SW480 cells. SW480 cells were treated with 5 and 10 μM of hinokitiol for 72 h and nuclear protein was isolated. Contents of 5hmC was measured using ELISA-based methylflash hydroxymethylated DNA quantification kit (a). The levels of 5mC and 5hmC were confirmed using flow cytometry analysis (b). All data are representative of three independent experiments performed in duplicate. The results were representative of three independent experiments. Data are the means ± SE of results from at least three independent experiments. (JPG 246 kb) [file 12860_2017_130_MOESM3_ESM.jpg]

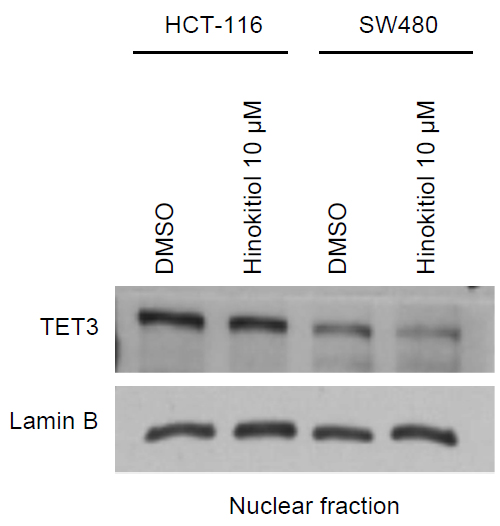

Supplement: Additional file 4: — Hinokitiol has no affect TET3 protein expression in colon cancer cells. Colon cancer cells (HCT-116 or SW480) were treated with 10 μM of hinokitiol for 72 h. Nuclear protein was extracted from the cells and then western blot analysis was performed with anti-TET3 and anti-Lamin B antibodies. Lamin B was used for loading control. Experiments were done twice and representative blots were shown. Cells treated with DMSO or 5-aza-dC were used as negative and positive controls, respectively. (JPG 102 kb) [file 12860_2017_130_MOESM4_ESM.jpg]
